# Detection of the mosquito-borne flaviviruses, West Nile, Dengue, Saint Louis Encephalitis, Ilheus, Bussuquara, and Yellow Fever in free-ranging black howlers (*Alouatta caraya*) of Northeastern Argentina

**DOI:** 10.1371/journal.pntd.0005351

**Published:** 2017-02-10

**Authors:** María A. Morales, Cintia M. Fabbri, Gabriel E. Zunino, Martín M. Kowalewski, Victoria C. Luppo, Delia A. Enría, Silvana C. Levis, Gladys E. Calderón

**Affiliations:** 1 Departamento Investigación, Instituto Nacional de Enfermedades Virales Humanas “Dr. Julio I. Maiztegui”, ANLIS, Pergamino, Argentina; 2 Instituto del Conurbano, Área Ecología Universidad Nacional de General Sarmiento, Buenos Aires, Argentina; 3 Estación Biológica de Usos Múltiples de Corrientes -CONICET (EBCo), Museo Argentino de Ciencias Naturales, Argentina; Duke-NUS GMS, SINGAPORE

## Abstract

Several medically important mosquito-borne flaviviruses have been detected in Argentina in recent years: Dengue (DENV), St. Louis encephalitis (SLEV), West Nile (WNV) and Yellow Fever (YFV) viruses. Evidence of Bussuquara virus (BSQV) and Ilheus virus (ILHV) activity were found, but they have not been associated with human disease. Non-human primates can act as important hosts in the natural cycle of flaviviruses and serological studies can lead to improved understanding of virus circulation dynamics and host susceptibility. From July–August 2010, we conducted serological and molecular surveys in free–ranging black howlers (*Alouatta caraya*) captured in northeastern Argentina. We used 90% plaque-reduction neutralization tests (PRNT_90_) to analyze 108 serum samples for antibodies to WNV, SLEV, YFV, DENV (serotypes 1and 3), ILHV, and BSQV. Virus genome detection was performed using generic reverse transcription (RT)-nested PCR to identify flaviviruses in 51 antibody-negative animals. Seventy animals had antibodies for one or more flaviviruses for a total antibody prevalence of 64.8% (70/108). Monotypic (13/70, 19%) and heterotypic (27/70, 39%) patterns were differentiated. Specific neutralizing antibodies against WNV, SLEV, DENV-1, DENV-3, ILHV, and BSQV were found. Unexpectedly, the highest flavivirus antibody prevalence detected was to WNV with 9 (8.33%) monotypic responses. All samples tested by (RT)-nested PCR were negative for viral genome. This is the first detection of WNV-specific antibodies in black howlers from Argentina and the first report in free-ranging non-human primates from Latin-American countries. Given that no animals had specific neutralizing antibodies to YFV, our results suggest that the study population remains susceptible to YFV. Monitoring of these agents should be strengthened to detect the establishment of sylvatic cycles of flaviviruses in America and evaluate risks to wildlife and human health.

## Author summary

Flaviviruses are responsible for a growing disease burden in Argentina and other countries in the Americas. Non-human primates, such as monkeys, can be important hosts in the natural cycle of several flaviviruses. Yellow Fever virus outbreaks occurred in Argentina during 2007–2009 in areas of Misiones and Corrientes provinces inhabited by black howlers (*Alouatta caraya*), a monkey that is highly susceptible to the virus. During 2010 we tested 108 black howlers from Northeastern Argentina for flaviviruses. Most of these animals were negative for Yellow Fever virus but had antibodies to several other flaviviruses. Unexpectedly, the highest specific neutralizing antibody prevalence was to West Nile Virus; these results represent the first evidence of West Nile Virus circulation in a new host in Argentina. Detection of Dengue virus antibodies in black howlers highlights the potential for establishment of a dengue sylvatic cycle, not yet demonstrated in the Americas. We call for strengthening the monitoring of flaviviruses to evaluate risk for wildlife and human health in the region.

## Introduction

Emerging and re-emerging diseases are one of the main threats to global public health; 60% of these diseases are zoonoses (diseases shared between humans and vertebrate animals) and the majority originated from wildlife [1]. The increasing incidence of these diseases is related to the intense ecological changes that occur at local, regional, and global scales. Several unexpected emergences of zoonotic flaviviruses worldwide were recently recognized. The introduction of West Nile virus (WNV) and Zika virus (ZIKV) into the New World [2,3] and the emergence of Japanese encephalitis virus in Australia a few prominent examples [4]. Currently there are 39 defined members of the mosquito-borne viruses of the genus *Flavivirus* [5]. They usually infect a variety of vertebrate and mosquito species. Some flaviviruses have a limited number of hosts and vectors, others replicate in many hosts and vectors. Some have an extremely widespread distribution; others are spatially restricted. The potential of flaviviruses to cause disease in humans is significant and they have a potential to induce losses in livestock or wild animals of economic and ecological importance.

Several of the most prominent and medically important mosquito-borne flaviviruses were detected in Argentina in recent years: Dengue virus (DENV), St. Louis encephalitis virus (SLEV), WNV and Yellow Fever virus (YFV). During 2016, Zika virus was also detected in Argentina with autochthonous circulation restricted to Tucuman Province [6]. Other flaviviruses circulating in Argentina include Bussuquara virus (BSQV) and Ilheus virus (ILHV), which have not yet been associated with human disease [7].

Dengue viruses have emerged as the most important human arboviral pathogens from non-human primate enzootic reservoirs to humans resulting in an urban endemic transmission cycle. In Africa and Southeast Asia the viruses have been maintained in a sylvatic cycle, most likely involving non-human primates as reservoirs. These cycles have not been recognized in South America, but serological studies have suggested a possible secondary amplification cycle involving mammals other than non-human primates. The question of whether mammals maintain DENV in enzootic cycles and can play a role in its reemergence in human populations remains to be answered [8, 9]. Argentina was free of dengue for more than 80 years before the disease was detected in 1998. However, in the last 18 years, indigenous DENV circulation has been reported in Northern and Central Argentina, representing a growing public health problem [6, 10, 11].

Since 2002 Argentina has experience the re-emergence of SLEV, with febrile illness and encephalitis outbreaks in humans, mainly in temperate areas of the country [12–14]. Genotypes II, III, V, and VII of SLEV were detected in mosquitoes and rodents [15, 16]. High SLEV antibody prevalence was demonstrated in black howlers in Argentina and southern Brazil but the role that primates could play in viral maintenance in nature is unknown [17, 18].

The isolation of WNV from equines in Argentina in 2006 was the first direct evidence of its circulation in the Southern Cone. Nucleotide sequences showed that the virus belonged to clade 1a of lineage 1 and clustered in a subclade with American strains isolated during 1999–2002 [19, 20]. Public health surveillance in Argentina detected sporadic human cases in 2006–2007 in five provinces of the northeast and central areas of the country(Chaco, Entre Ríos, Formosa, Santa Fé, and Córdoba Provinces) but the impact on animal and human public health was considerably lower than in the northern hemisphere until now [21, 22]. Detection of WNV in resident birds in 2005–2006 suggested that it was introduced into Argentina and maintained naturally in enzootic foci where numerous bird species from many families were exposed [23]. The transmission cycle of WNV commonly involves birds and *Culex* mosquitoes, but it is not well known in Argentina. Recent studies of vector competence showed that Argentine *Culex* are competent vectors, but they were characterized as moderately efficient vectors of WNV and less susceptible to infection than US mosquito strains [24].

Yellow fever is an infectious disease that remains endemic or enzootic in rainforests of South America and sub-Saharan Africa. The sylvatic yellow fever cycle is maintained by viral circulation between monkeys and diurnally active mosquitoes that breed in tree holes in the forest canopy. Many species of non-human primates are hosts of this cycle. The species most commonly involved in virus transmission are New World monkeys of the genera *Cebus*, *Alouatta*, and *Callithrix*. The susceptibility of monkeys to lethal infections of YFV in America has been considered a major indicator for enzootic disease outbreaks in forest areas [25–27]. Sylvatic cases of yellow fever in humans were often preceded by epizootics in animals in Brazil and Argentina [28, 29]. Black howlers inhabit the Chaco and Pantanal ecoregions in Brazil, Paraguay, Bolivia, and north-northeastern Argentina, a small portion of the Atlantic Forest in Misiones Province, Argentina, and the state of Rio Grande do Sul, Brazil [30–32]. Epizootics were reported in Argentina during 2007–2009 in Misiones and Corrientes Provinces where four native species of monkeys live, including the black howler (*Alouatta caraya*) [33, 34]. This species has the southernmost distribution of all primate species in the Neotropics, reaching latitude 29°S. In Argentina, black howlers inhabit a complex forest consisting of humid Chaco forest, savannas, gallery forest, and flooded forest (Chaco, Formosa, Corrientes, and Santa Fe provinces). Their populations in the upper Paraná Atlantic Forest are fragile and recurrence of YFV circulation or other pathogens could be harmful to the species maintenance [35, 36].

Viruses and viral disease outbreaks play an ecological role increasingly recognized in populations of wild animals. At least 27 viruses have been reported to infect both humans and wild primates and most of them are classified as emerging threats to human populations [37, 38]. The rapid expansion of human activities into habitats of primates has resulted in increased potential for exchange of pathogens, creating challenges for biodiversity conservation and global health. The role of many wildlife species as reservoirs for arthropod-borne viral pathogens is poorly understood. Virus-specific antibody detection in a wildlife species could indicate a reservoir host or a species that could serve as a sentinel for virus activity in nature.

Due to the impact of recent yellow-fever epidemics, there was special concern about the status of the black howler, which is the monkey species most affected by epizootics in Argentina. We conducted serological and molecular tests to detect flavivirus circulation in free-living black howlers in Northeast Argentina in 2010.

## Materials and methods

### Study sites

**Fig 1:**
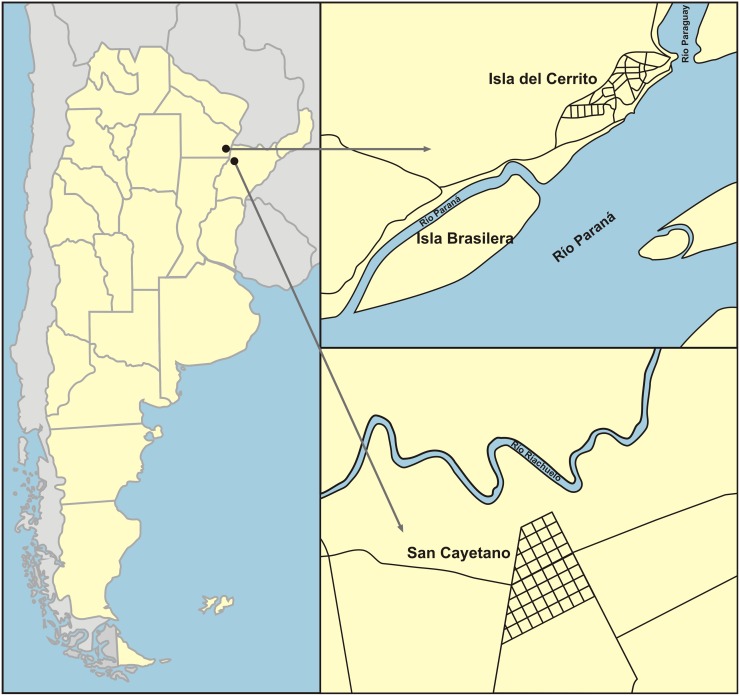
Study sites in Chaco and Corrientes provinces, Northeastern Argentina.

The study was carried out in July–August 2010 in San Cayetano (SC), Corrientes province (27 ° 34 'S, 58 ° 41' W); Isla del Cerrito (IC) (27° 17´S, 58° 37´W), and Isla Brasilera (IB), Chaco province (27 ° 20 'S, 58 ° 40' W) in northeastern Argentina (Fig 1). San Cayetano is a savanna with degraded and fragmented semi-deciduous forest. Forest fragments have been modified by deforestation, cattle introduction, the reuse of land for plantations, and burning trees allowing humans and monkeys to live in close association [39]. Isla del Cerrito and Isla Brasilera are at the confluence of the Paraguay and Paraná Rivers and are characterized by continuous flooded forest. Sites were classified following two criteria: areas where primate habitat overlapping human populations and agricultural activities (SC and IC) and wild areas where human contact is rare (IB).

### Sample collection

Captured black howlers were immobilized with methomidine hydrochloride combined with ketamine hydrochloride, administered via a dart driven by compressed air. To maintain body temperature at optimum conditions, animals were covered with blankets and warm water bottles were placed with them throughout the procedure. From 109 captured black howlers, we collected 108 blood samples. Distribution by provinces was 51 (51.5%) captures in Chaco and 58(48.5%) in Corrientes. Sex, weight, and measurements were recorded [40]. Of the animals studied, 43 (39.8%) were female and 65 (60.2%) were male; 85% were adults and the rest were immature. Blood samples were obtained by puncture of the femoral vein. After evaluation of their health, each animal was transferred to the exact site of capture and observed until it moved into the habitat. Blood samples were centrifuged for at least 10 min at 2000 x g for serum separation and stored in liquid nitrogen in the field. At the laboratory, samples were frozen at -80°C until tested.

### Ethics statement

This research complied with the Code of Best Practices for Field Primatology (International Primatological Society), the guidelines for the ethical treatment of primates (IACUC protocol 09267) and the laws of Argentina (through Dirección de Recursos Naturales, Provincia de Corrientes and Dirección de Fauna, Provincia de Chaco, plus approval of the National Institute of Human Viral Diseases, Dr. Julio I. Maiztegui, Ethics Committee for Biomedical Research. The animal capture and identification techniques were designed to be less invasive to preserve the welfare of the animals and relieve potential stress.

### Plaque-reduction neutralization tests (PRNT)

Serum samples were heat inactivated at 56°C for 30 min. Two-fold serial dilutions from 1:10 to 1:2560 of each sample were incubated with 100 plaque-forming units (PFU) of WNV (strain ChimeriVax TM WNV), SLEV (strain ChimeriVax TM SLEV), DENV-1 (strain Hawaii), DEN-3 (strain H87), YFV (vaccine strain 17D-YEL), ILHV (Original) and BSQV [41]. Vital dye neutral red was used at 5% for plaque visualization. Plaques were counted and titers were calculated and expressed as the reciprocal of the serum dilution yielding a ≥90% reduction in PFU on Vero cells (PRNT_90_). Titers ≥10 were considered positive. Monotypic or heterotypic patterns were differentiated according to whether the animal was positive to one or several flaviviruses, respectively. In heterotypic patterns, interpretation of PRNT data was as follows: animals with a neutralizing antibody titer (PRNT_90_) ≥ four-fold higher than the other flavivirus titers were considered positive for antibody to that virus.

Animals with neutralizing antibody titers against multiple viruses without four-fold difference in titer were considered flavivirus antibody positive with no specific virus identified and labeled as “undetermined” flavivirus.

### Viral genome detection

The molecular approach was performed on sera from 27 animals that were PRNT_90_ antibody negative for all the flaviviruses in our panel and 24 animals that were YFV antibody negative. Viral RNA was extracted from 140 uL of serum using QIAamp viral RNA extraction kit (Qiagen, Inc., Valencia, California, USA) and then generic reverse transcription (RT)-nested PCR was used to identify flaviviruses. This procedure was used to amplify a specific 143-bp fragment of the NS5 gene [42]. The amplified products were visualized by ethidium bromide staining after electrophoresis on a 2.0% high-resolution agarose gel.

## Results

Of the 108 black howlers studied, 64.8% (70/108) had evidence of past flavivirus infection. Monotypic (13/70, 19%) and heterotypic (27/70, 39%) patterns were differentiated. The remaining 42% of antibody-positive animals was classified as undetermined for virus identification.

We identified specific neutralizing antibodies against WNV, SLEV, DENV-1, DENV-3, ILHV and BSQV. Antibody prevalences were 22.2% (24/108 with 9 monotypic responses) for WNV, 10.2% (11/108) for SLEV, 1.85% (2/108, 100% monotypic) for DENV (1/108 DENV-3 and 1/108 DENV-1), 0.93% (1/108, monotypic) for ILHV, and 0.93% (1/108, monotypic) for BSQV. Distribution of PRNT_90_ titers are shown in Table 1 (monotypic pattern) and Table 2 (heterotypic pattern). The WNV antibody titers (and frequency) in antibody-positive animals with monotypic pattern were: 20 (1); 40 (4), 80(3), and 160 (1) (Table 1). The WNV antibody titer distribution in animals with heterotypic pattern was: 40 (6); 80 (2); 160 (2); 320 (4), >1280 (1) (Table 2).

**Table 1:** Distribution of PRNT_90_ titers for 7 flaviviruses among 13 positive black howlers with monotypic immune pattern.

Animal identification	PRNT_90_ Titer							Result Interpretation
	DENV-1	DENV-3	SLEV	WNV	YFV	ILHV	BSQV	
PA07001	<10	<10	<10	**80**	<10	<10	<10	WNV
PA07007	<10	<10	<10	**80**	<10	<10	<10	WNV
PA07008	<10	<10	<10	**80**	<10	<10	<10	WNV
PA07021	<10	**20**	<10	<10	<10	<10	<10	DENV-3
PA07036	<10	<10	<10	**40**	<10	<10	<10	WNV
PA07075	**80**	<10	<10	<10	<10	<10	<10	DENV-1
PA07079	<10	<10	<10	**40**	<10	<10	<10	WNV
PA07082	<10	<10	<10	**40**	<10	<10	<10	WNV
PA07083	<10	<10	<10	**160**	<10	<10	<10	WNV
PA07092	<10	<10	<10	**40**	<10	<10	<10	WNV
PA07099	<10	<10	<10	<10	<10	<10	**20**	BSQV
PA07103	<10	<10	<10	<10	<10	20	<10	ILHV
PA07108	<10	<10	<10	**20**	<10	<10	<10	WNV

**Table 2:** Distribution of PRNT_90_ titers for 7 flaviviruses among 27 positive black howlers with heterotypic immune pattern.

Animal Identification	PRNT_90_ Titer							Result Interpretation
	DENV-1	DENV-3	SLEV	WNV	YFV	ILHV	BSQV	
PA07019	10	10	**160**	20	<10	10	<10	SLE V
PA07030	<10	40	<10	**320**	<10	10	40	WNV
PA07035	<10	<10	**40**	10	<10	<10	<10	SLEV
PA07039	<10	<10	80	**320**	<10	10	<10	WNV
PA07040	<10	<10	<10	**80**	<10	20	10	WNV
PA07043	20	<10	**640**	10	<10	<10	<10	SLEV
PA07052	80	40	**320**	80	<20	20	10	SLEV
PA07073	<10	<10	10	**40**	<10	<10	<10	WNV
PA07076	80	10	**320**	80	<10	10	10	SLEV
PA07077	40	10	**160**	20	<10	<10	<10	SLEV
PA07078	10	20	**80**	<10	<10	<10	<10	SLEV
PA07084	10	10	10	**320**	<10	10	10	WNV
PA07085	<10	<10	10	**40**	<10	10	10	WNV
PA07088	<10	<10	10	**40**	<10	<10	<10	WNV
PA07091	10	20	**>1280**	20	<10	<10	<10	SLEV
PA07100*	<10	<10	40	**160**	10	<10	<10	WNV
PA07102	<10	<10	<10	**80**	<10	20	<10	WNV
PA07104*	20	20	**640**	160	20	40	20	SLEV
PA07105	<10	<10	10	**40**	<10	<10	<10	WNV
PA07109	<10	<10	10	**40**	<10	<10	<10	WNV
PA07112	10	20	40	**>1280**	<10	>20	10	WNV
PA07113	20	10	20	**320**	<10	10	10	WNV
PA07117	10	10	**80**	10	<10	<10	<10	SLEV
PA07118	<10	10	**40**	10	<10	<10	<10	SLEV
PA07121	10	10	<10	**40**	<10	<10	<10	WNV
PA07016	<10	<10	<10	**160**	<20	20	<10	WNV
PA07049	<10	<10	**40**	<10	<10	10	<10	SLEV

*Black howler with neutralizing antibodies against YFV.

DENV-1, Dengue virus serotype 1; DENV-3, Dengue virus serotype 3; SLEV, Saint Louis encephalitis virus; WNV, West Nile virus; YFV, Yellow fever virus; ILHV, Ilheus virus and BSQV, Bussuquara virus.

There were no statistically significant differences in the prevalences of infection for each flavivirus between sexes or among study sites or habitat classes. When antibody distribution was analyzed by age, a statistical difference was observed only for WNV antibody prevalence in adult black howlers (p = 0.0075).

In 30 of 70 animals (43%) results were inconclusive because of neutralizing antibody titers against multiple viruses without fourfold difference; those were considered positive for an undetermined flavivirus (Table 3). We observed different reactivity among these: 73% (22/30) for WNV, 61% (18/30) for SLEV, 61% (18/30) for ILHV, 53% DENV-3(16/30), 47% (14/30) for DENV-1, 33% (10/30) for BSQV and 7% (2/30) for YFV.

**Table 3:** Distribution of PRNT_90_ titers for 7 flaviviruses among 30 black howlers positive for several flaviviruses without four-fold differences (undetermined flavivirus).

Animal Identification	PRNT_90_ Titer							Undetermined for
	DENV-1	DENV-3	SLEV	WNV	YFV	ILHV	BSQV	
PA07011	<10	<10	**10**	<10	<10	<10	<10	SLEV **
PA07014	<10	<10	**640**	**640**	<10	<10	<10	SLEV, WNV
PA07017	<10	<10	<10	<10	<10	**10**	<10	ILHV**
PA07018	<10	<10	<10	**20**	<10	**40**	<10	WNV,ILHV
PA07027	<10	**10**	<10	<10	<10	<10	<10	DENV-3 **
PA07037	<10	**10**	<10	**10**	<10	<10	<10	DENV-3, WNV
PA07038	<10	**10**	<10	**10**	<10	**20**	**20**	DENV-3,WNV, ILHV
PA07042	<10	<10	<10	10	<10	<10	<10	WNV **
PA07045	<20	<20	**160**	**80**	<20	<10	<10	SLEV, WNV
PA07050	**20**	<10	<10	<10	<10	**10**	<10	DENV-1,ILHV
PA07054	**40**	**40**	<20	<20	<20	<10	<10	DENV-1, DENV-3
PA07055	**40**	**20**	<20	<20	<20	<10	<10	DENV-1, DENV-3
PA07072	**80**	**40**	**1280**	**1280**	20*	**80**	**10**	SLEV,WNV, DENV-1,DENV-3,ILHV,BSQV, YFV
PA07074	**40**	<10	**80**	**40**	<10	10	<10	DENV-1, SLEV,WNV,ILHV
PA07080	**10**	**20**	**160**	**640**	<10	**320**	**20**	DENV-1,DENV-3,SLEV,WNV, ILHV, BSQV
PA07081	<10	**20**	**160**	**320**	<10	**10**	**80**	DENV-3,SLEV, WNV, ILHV, BSQV
PA07086	**10**	**10**	**320**	**640**	<10	**10**	**40**	DENV-1,DENV-3,SLEV,WNV,ILHV, BSQV
PA07094	**10**	**10**	**320**	**320**	<10	**20**	**20**	DENV-1,DENV-3,SLEV, WNV, ILHV, BSQV
PA07095	<10	<10	**10**	**40**	<10	**20**	**10**	WNV,SLEV, ILHV, BSQV
PA07097	<10	**20**	**20**	**40**	<10	**10**	<10	DENV-3, SLEV, WNV, ILHV
PA07101	**40**	**20**	<10	**20**	<10	**10**	<10	DENV-1,DENV-3, WNV, ILHV
PA07106	**20**	**10**	**40**	**40**	<10	**20**	**10**	DENV-1,DENV-3, SLEV,WNV,ILHV,BSQV
PA07107	**10**	**10**	**80**	**40**	<10	**20**	**10**	DENV-1, DENV-3, SLEV,WNV, ILHV, BSQV
PA07110	**10**	<10	**80**	**40**	10*	**40**	**40**	DENV-1, SLEV,WNV, ILHV, BSQV, YFV
PA07111	<10	<10	**40**	**40**	<10	**10**	<10	SLEV, WNV, ILHV
PA07114	<10	<10	<10	**10**	<10	<10	<10	WNV **
PA07115	**10**	**20**	**40**	<10	<10	**10**	<10	DENV-1, DENV-3, SLEV, ILHV
PA07116	<10	<10	<10	**10**	<10	<10	<10	WNV **
PA07119	<10	<10	**20**	**10**	<10	<10	<10	SLEV, WNV
PA07120	**10**	**10**	<10	<10	<10	<10	<10	DENV-1, DENV-3

*Black howler with neutralizing antibodies against YFV.

** Without 4-fold difference because the lowest dilution studied was 1:10

DENV-1, Dengue virus serotype 1; DENV-3, Dengue virus serotype 3; SLEV, Saint Louis encephalitis virus; WNV, West Nile virus; YFV, Yellow fever virus; ILHV, Ilheus virus and BSQV, Bussuquara virus.

For molecular studies, we selected serum samples from 51 animals. These were 27 animals without flavivirus antibodies and 24 that were YFV antibody negative. All animals analyzed by (RT)–nested PCR were negative for flavivirus genome.

## Discussion

Our goal was to understand the potential role of free-living black howlers as hosts in the natural cycles of flaviviruses in Argentina. The PRNT_90_ used to identify specific antibodies in black howler serum samples indicated a variable prevalence for one or more of six of the seven flaviviruses tested including WNV, SLEV, DENV-1, DENV-3, ILHV, and BSQV. No specific immune response to YFV was detected.

Thirty-four groups of black howlers have been identified in the study area, in about 3,000 ha, several of these groups have been under behavioral study since 2000 [43, 44] in undisturbed forest and in forest fragmented by human activities. These regions provide favorable conditions for the occurrence of outbreaks or for enzootic maintenance of arthropod-borne diseases.

Yellow fever outbreaks occurred near this region in November 2007–October 2008, seriously affecting the populations of two howler monkey species: the brown howler (*Alouatta guariba clamitans*) and the black howler. In these epizootics, the deaths of 65 monkeys were detected in Misiones and Corrientes provinces [33]. Herein we focused on the prevalence of infection in black howlers with those flaviviruses of recognized public health impact in Argentina (YFV,DENV, SLEV, and WNV). We also included ILHV and BSQV because of their known occurrence in Argentine wildlife.

All flaviviruses are serologically related, which can be demonstrated by binding assays suchas ELISA and by hemagglutination-inhibition tests using polyclonal and monoclonal antibodies. The PRNT_90_ is one of the most specific test available often used to define several serocomplexes of more closely related flaviviruses. The viral panel was employed to evaluate serological cross-reactions. To increase specificity, we selected a conservative threshold of 90% for PRNT. According with 9^th^Report of the International Committee of Taxonomy of Viruses [5], WNV and SLEV have been placed together in the Japanese encephalitis group. DENV-1, DENV-2, DENV-3 and DENV-4 compose the dengue virus group. Ilheus virus was included in the Ntaya virus group and BSQV in Aroa virus group. Yellow fever virus is the prototype of the genus *Flavivirus* and is in its own virus group. Analysis of serological results requires careful evaluation especially when co-circulation of multiple flaviviruses is expected, as in Argentina.

We found different immune patterns in the positive animals: 19% monotypic, 39% heterotypic, and 43% classified as undetermined for viral identification according to our criteria. We included 6 animals in the last group which had only titer of 10 for WNV (3), SLEV (1), DENV-3 (1) and ILHV (1); we couldn´t use 4 fold criteria because the lowest dilution studied was 1:10. When individuals with no previous exposure to a flavivirus are infected with one, a monotypic response to the infecting virus is demonstrated in serological tests, such as PRNT, and the etiologic agent can be accurately identified. Interpretation of heterotypic patterns is complex and 4-fold difference in titers could be a limited criterion to provide distinction of the most recent infection. The antibody response in sequential experimentally infected animals illustrates the difficulty for a serologic diagnosis of WNV infection in animals (or humans) with preexisting flavivirus immunity [45]. Sequential infections with other flavivirus elicit strong cross-reactive anamnestic responses, which may confer immunity, especially within members of the same serogroup [46]. For example, sequential infections SLEV-WNV or WNV-SLEV would present different immune patterns that hinder serological differentiation of each [45]. Monotypic serologic responses were the most reliable as these samples reacted with only one of the 7 viruses employed in the tests. Selection of the virus in the panel is critically important. Future similar studies in Argentina might also include ZIKV.

Prevalence of antibody to WNV antibody was the highest among the flaviviruses evaluated. The monotypic WNV prevalence was 8.33% (9/108) and represents the clearest serologic evidence of WNV activity in a new host in Argentina. We found WNV-positive black howlers at all sampling sites, demonstrating a widespread distribution in the study region.

Higher WNV antibody titers were detected in the group with a heterotypic pattern. This type of response could reflect sequential infections or cross reactions originated by the higher antibody titers. We also detected WNV reactivity in 22 black howlers in the group classified as undetermined flavivirus, thus increasing the WNV antibody prevalence. Because of conservation considerations, we avoided capturing immature individuals or pregnant females. Thus, the higher prevalence in adults may reflect this capture bias. However, we should not rule out the possibility that this result could be due to undetected circulation of WNV in Argentina for some time before its isolation in 2006 [20, 23].

Prevalence of detectable antibody to SLEV in our study was 11% (12/108) without monotypic reaction. This value would be increased if we considered the animals that were SLEV positive among the group classified as “undetermined flavivirus”. Investigations in black howlers from this zone in 2001 confirmed infections with SLEV with prevalences of 35% (by hemagglutination test) and 32% (by PRNT). Studies in 2004–2005 demonstrated SLEV antibody prevalence (12% by hemagglutination test, 2% by mouse neutralization test) in free ranging black howlers in the upper Parana River basin in southern Brazil [17,18]. St. Louis encephalitis virus is endemically established in Argentina and was recognized in several human encephalitis outbreaks in the last decade [12,13, 14]. Our results indicate that SLEV and WNV could have been co-circulating within the region complicating the interpretation of serologic tests.

Only four animals had low neutralizing antibody titers for YFV despite recent epizootics, two of those had specific heterotypic reactions for WNV and SLEV and the others were in the positive group for undetermined flavivirus. These results could represent cross-serologic reaction or past infection. On the other hand, 51 animals were negative for viral genome. We did not detect YFV circulation in black howlers. This small and fragmented population suffers habitat destruction, hunting pressure, and cyclical yellow fever and thus, is at risk of disappearing in the long term [47]. The low antibody prevalence detected suggests that the population remains susceptible to YFV. An interesting aspect would be to know if preexisting antibodies for other flaviviruses could play a protective role in future YFV infections.

We detected monotypic reactions in PRNT_90_ against DENV-3 (0.93%) and DENV-1 (0.93%). Titers obtained were low but serum samples were previously heat inactivated to eliminate nonspecific reactions. These dengue serotypes were selected because their previous circulation was confirmed in human cases detected in Misiones, Corrientes, and Chaco provinces in 2000–2008. Besides, 47% of the animals were positive to DENV-1 and 53% for DENV-3 in the group of animals labeled us undetermined. As we mentioned previously, these results could be originated by cross reaction or they could represent limited spillback through contact with human environment, but we have to consider that other studies suggest the presence of sylvatic DENV in the Americas [48, 49, 50]. The establishment of a derived sylvatic cycle, as has happened with YFV in the Americas, will hinder the control of DENV in Latin America. Detection of DENV antibodies in black howlers in Northern Argentina underscores the importance of continuing the surveillance of these flaviviruses in non-human primate populations.

Ilheus virus is believed to be maintained in zoonotic cycles between birds and mosquitoes and has been isolated in Central and South America primarily from mosquitoes but also from sentinel monkeys and birds [51]. There are few reports of isolation of ILHV from humans in Central and South America with symptoms ranging from subclinical to severe febrile disease. Mild unspecific symptoms, brief viremia, and lack of laboratory screening techniques in situ are some of the impediments to diagnosis of ILHV infection in disease-endemic areas. The situation is similar for BSQV. We detected low specific prevalences to ILHV and BSQV antibodies, but higher among the animals found positive for an undetermined flavivirus.

Our work evidences circulation of WNV, SLEV, DENV-1, DENV-3, ILHV, and BSQV in wild, non-human primate populations of Corrientes and Chaco provinces, Argentina. Future studies might include ZIKV detection, which has been recognized in South America since 2015. To our knowledge this is the first detection of WNV-specific antibodies in black howlers from Argentina and the first report in free-ranging non-human primates from Latin America. Additionally, our results show that our study population remains susceptible to YFV as no specific neutralizing antibodies were detected. The black howler population remains tentative in the upper Paraná Atlantic Forest. Recurrence of YFV circulation or other pathogens could be detrimental to the population’s existence. Improved monitoring of these agents is needed to evaluate risk to wildlife and human health in the region.

## References

[1] Jones KE, Patel NG, Levy MA, Storeygard A, Balk D, Gittleman JL, Daszak P. Global trends in emerging infectious diseases. Nature. 2008; 451(7181):990–993. 10.1038/nature06536 18288193 PMC5960580

[2] Petersen LR and Roehrig JT. West Nile virus: a reemerging global pathogen. Emerg Infect Dis. 2001; 7(4): 611–614. 10.3201/eid0704.010401 11585520 PMC2631751

[3] Petersen LR, Jamieson DJ, Powers AM, Honein MA. Zika virus. N Engl J Med. 2016; 374:1552–63. 10.1056/NEJMra1602113 27028561

[4] Mackenzie JS, Gubler DJ, Petersen LR. Emerging flaviviruses: the spread and resurgence of Japanese encephalitis, West Nile and dengue viruses. Nat Med. 2004; 10, 98–109. 15577938 10.1038/nm1144

[5] King A.M.Q., Adams M.J., Carstens E.B., and Lefkowitz E.J. (Eds.). In: Family Flaviviridae Virus Taxonomy: Ninth Report of the International Committee on Taxonomy of Viruses. Elsevier Academic Press, San Diego, CA, 2012

[6] Integrated Epidemiological Surveillance Bulletin, Argentinean Ministry of Health, N321-SE31, 2016. http://www.msal.gob.ar/index.php/home/boletin-integrado-de-vigilancia

[7] Sabattini M. S., Aviles G., and Monath T. P.. Historical, epidemiological and ecological aspects of arboviruses in Argentina: Togaviridae, Alphavirus In: Travassos da Rosa A. P. A., Vasconcelos P. F. C., and Travassos da Rosa J. F. S. (eds), An Overview of Arbovirology in Brazil and Neighboring Countries, InstitutoEvandro Chagas, Belem, Brazil, 1998.

[8] Vasilakis N, Cardosa J, Hanley KA, Holmes EC and Weaver SC. Fever from the forest: prospects fo the continued emergence of sylvatic dengue virus and its impact on public health. Nature Rev Microbiol. 2011; 9: 532–541. 21666708 10.1038/nrmicro2595 PMC3321645

[9] De Thoisy B, Lacoste V, Germain A, Muñoz-Jordán J, Colón C, Mauffrey JF, Delaval M, Catzeflis F, Kazanji M, Matheus S, Dussart P, Morvan J, Setién AA, Deparis X, Lavergne A. Dengue infection in neotropical forest mammals. Vector Borne Zoonotic Dis. 2009; 9(2):157–70. 10.1089/vbz.2007.0280 18945183

[10] Avilés G, Paz MV, Rangeon G., Ranaivoarisoa MY, Verzeri N, Roginski S, Baroni P, Enria DA.Laboratory Surveillance of Dengue in Argentina, 1995–2001. Emerg. Infect. Dis. 2003; 9(6): 738–742 10.3201/eid0906.020483 12781019 PMC3000136

[11] Enría, DA and Morales, MA. Dengue. In: Virología Clínica. Guadalupe Carballal y José Raúl Oubiña (eds), 4 th. Ed, Corpus, 2015.

[12] Spinsanti LI, Díaz LA, Glatstein N, Arselán S, Morales MA, Farías AA, Fabbri C, Aguilar JJ, Ré V, Frías M, Almirón WR, Hunsperger E, Siirin M, Da Rosa AT, Tesh RB, Enría D, Contigiani M. Human outbreak of St. Louis encephalitis detected in Argentina, 2005.J ClinVirol. 2008; 42(1):27–33. 10.1016/j.jcv.2007.11.022 18249032

[13] Seijo A, Morales A, Poustis G, Romer Y, Efron E, Vilora G, LLoveras S, Giamperetti S, Puente T, Monroig J, Luppo V, Enria DA. Brote de Encefalitis de San Luis en el Area Metropolitana de Buenos Aires. Medicina (B. Aires). 2011; 71: 211–217. 21745768

[14] Fabbri CM, Morales MA, Luppo VC, CappatoBerger F, Balanitro B, Manrique M, Fierro F, Goenaga S, Enria DA, Levis SC. Brote de Encefalitis de San Luis en la Provincia de San Juan, Argentina, 2011.Rev Argent Microbiol.2011; 1 (43): 89.

[15] Diaz LA, Ré V, Almirón WR, Farías A, Vázquez A, Sanchez-Seco MP, Aguilar J, Spinsanti L, Konigheim B, Visintin A, Garciá J, Morales MA, Tenorio A, Contigiani M. Genotype III Saint Louis encephalitis virus outbreak, Argentina, 2005.Emerg Infect Dis. 2006 11;12(11):1752–4. 17283629 10.3201/eid1211.060486 PMC3372344

[16] Díaz LA, Albrieu-Llinás G, Vázquez A, Tenorio A, Contigiani MS. Silent circulation of St. Louis Encephalitis virus prior to an encephalitis outbreak in Córdoba, Argentina. PLoS Negl Trop Dis.2005; 2012; 6: 1489. 10.1371/journal.pntd.0001489 PMC3269431 22303490

[17] Contigiani MS, Fernández C, Spinsanti LI, Díaz GE. Prevalence of Flavivirus antibodies in *Alouatta caraya* primate autochthonous of Argentina. Medicina (B. Aires). 2000; 60(3):348–50. 11050814

[18] Svoboda WK, Martins LC, Molanski LS, Shiozawa MM, Spohr KAH, et al Serological evidence for Saint Louis encephalitis virus in free-ranging New World monkeys and horses within the upper Paraná River basin region, Southern Brazil. Rev Soc Bras Med Trop. 2014; 47: 280–286. 25075477 10.1590/0037-8682-0083-2014

[19] Morales MA, Barrandeguy M, Fabbri C, Garcia J, Vissani A., Trono K, Gutierrez G, Pigretti S, Menchaca H, Garrido N, Taylor N, Fernandez F, Levis S. y D Enría.Isolation of West Nile virus (WNV) fromequines in Argentina, 2006. Emerg. Infect. Dis. 2006, 12(10): 1559–1561. 17176571 10.3201/eid1210.060852 PMC3290965

[20] Fabbri CM, García JB, Morales MA, Levis S, Enría DA, Lanciotti RS. Complete genome sequences and phylogenetic analysis of two West Nile virus strains isolated from equines in Argentina in 2006 could indicate an early entrance of the virus in the southern cone. Vector-Borne and Zoonotic Dis.2014; 14 (11): 794–800. 10.1089/vbz.2014.1588 25409270

[21] Artsob H, Gubler DJ, Enria DA, Morales MA, Pupo M, Bunning ML and Dudley JP. West Nile Virus in the New World: Trends in the Spread and Proliferation of West Nile Virus in the Western Hemisphere. Zoonoses and Public Health. 2009; 56: 257–428. 19486320 10.1111/j.1863-2378.2008.01207.x

[22] Enría, DA and Morales, MA. Virus del Nilo Occidental. In: “Virología Clínica”. Guadalupe Carballal y José Raúl Oubiña, p 619–621. Corpus, 4 th. Edition, 2015

[23] Diaz LA, Komar N, Visintin A, DanturJuri MJ, Stein M, Lobo Allende R, Spinsanti L, Konigheim B, Aguilar J, Laurito M, et al West Nile Virus in birds, Argentina. Emerg. Infect. Dis. 2008;14:689–691. 10.3201/eid1404.071257 18394305 PMC2570931

[24] Micieli MV, Matacchiero AC, Muttis E, Fonseca DM, Aliota MT, Kramer LD. Vector competence of Argentine mosquitoes (Diptera: Culicidae) for West Nile virus (Flaviviridae: Flavivirus). J Med Entomol. 2013; 50(4):853–62. 23926785 10.1603/me12226 PMC3932752

[25] Ott-Joslin JE. Viral diseases in nonhuman primates In: Fowler M, editor. Zoo and wild animal medicine. United States of North America: W.B. Saunders Co, p 674–697, 1986.

[26] Richter CB, Lehner NDM, Henrickson RV. Primates In: Fox JG, Cohen BJ, Loew FM, editors. Laboratory animal medicine. San Diego, United States of North America: Academic Press Inc, p 287–383, 1984.

[27] Vasconcelos PFC. Febre amarela. RevSocBrasMedTrop. 2003; 36:275–293. 10.1590/s0037-86822003000200012 12806465

[28] Almeida MAB, Santos E, Cardoso JC, Fonseca DF, Noll CA, Silveira VR, et al Yellow fever outbreak affecting *Alouatta* populations in Southern Brazil (Rio Grande do Sul). Ame J Primatol. 2012; 74:68–76 10.1002/ajp.21010 22020690

[29] Morales MA y Fabbri CM. Diagnóstico de las infecciones por el virus de la Fiebre Amarilla (YFV): Experiencia durante la reemergencia de Fiebre Amarilla Selvática en Argentina, 2008–2009. In: Temas de Zoonosis VI, Asociación Argentina de Zoonosis,pp157-166,2014, Argentina.

[30] Rumiz DI. *Alouatta caraya*: population density and demography in Northern Argentina.Am J Primatol. 1999; 21:279–294. 10.1002/ajp.1350210404 31963967

[31] Di Bitetti MS. Outlook for Primate Conservation in Misiones In: The State of the Hotspots: The Atlantic Forest of South America: Biodiversity Status, Threats, and Outlook. Galindo Leal C & De GuzmaoCamara I (eds.), Island Press, Center for Applied Biodiversity Science at Conservation International, Washington, 2013

[32] Leiroz Codenotti T, Martins Da Silva V, de Albuquerque VJ, Camargo EV, Martins Silveira RM. 2002. Distribuc-a˜o e situac-a˜o actual de conservac-a˜o de Alouatta caraya (Humboldt, 1812) no Rio Grande do Sul, Brazil. Neotropical Primates. 2002; 10(3):132–141.

[33] Holzmann I, Agostini I, Areta JI, Ferreyra H, Beldomenico P, Di Bitetti MS. Impact of Yellow Fever outbreaks on two howler monkey species (*Alouattaguaribaclamitans*and *A*. *caraya*) in Misiones, Argentina. American Journal of Primatology. 2010;71:1–6. 10.1002/ajp.20796 20095025

[34] Goenaga S, Fabbri C, Duenas JCR, Gardenal CN, Rossi GC, Calderon G, Morales MA, Garcia JB, Enria DA, Levis S. *Isolation of Yellow Fever Virus from mosquitoes in Misiones Province*, *Argentina*. Vector-borne and Zoonotic Diseases. 2012; 12(11):1–8. 23025694 10.1089/vbz.2011.0730

[35] Agostini I, Aprile G, Baldovino MC, Brividoro M, Di Bitetti M, Fantini L, Fernández VA, Fernández-Duque E, Holzmann I et al Orden Primates In: *Libro rojo de mamíferos amenazados de la Argentina*. Ojeda RA, Chillo V, DiazIsenrath GB, Eds; Sociedad Argentina para el Estudio de los Mamíferos (SAREM), pp. 81–86, 2012.

[36] Pedersen A. B., Altizer S., Poss M., Cunningham A. A. & Nunn C. L. Patterns of host specificity and transmission among parasites of wild primates. Int. J. Parasitol. 2005; 35, 647–657. 10.1016/j.ijpara.2005.01.005 15862578

[37] Nunn C.L. and Altizer S.M. Infectious Diseases in Primates: Behavior, Ecology and Evolution. Oxford University Press, Series in Ecology and Evolution, 2006.

[38] Pedersen AB and Davies TJ. Phylogeny and geography predict pathogen community similarity in wild primates and humans.Proc Biol Sci. 2008; 275(1643): 1695–1701. 10.1098/rspb.2008.0284 18445561 PMC2602822

[39] Zunino GE, Kowalewski MM. Primate research and conservation in northern Argentina: the field station Corrientes (EstaciónBiológica de UsosMúltiples—EBCo). Tropical ConservationScience. 2008; 1(2):140–150. Available online: tropicalconservationscience.org

[40] Coppo J., &Resoagli E. Etapas de crecimiento en monos caraya. Facena 1978; 2, 29–39.

[41] Russel PK, Nisalak A, Sukhavachana P, Vivona S. A plaque reduction test for dengue virus neutralizing antibodies. J Immunol.1967;99:291–6. 6031202

[42] Sánchez-Seco MP, Rosario D, Domingo C, Hernández L, Valdes K, Guzman MG, Tenorio A. Generic RT-nested-PCR for detection of flaviviruses using degenerated primers and internal control followed by sequencing for specific identification. J Virol Methods. 2005; 126:101–9. 10.1016/j.jviromet.2005.01.025 15847925

[43] Zunino GE, Kowalewski MM, Oklander LI, Gonzalez V. Habitat fragmentation and population trends of the black and gold howler monkey (Alouattacaraya) in a semideciduous forest in northern Argentina. Am J Primatol. 2007; 69: 966–975. 10.1002/ajp.20389 17358009

[44] Kowalewski MM. Patterns of affiliation and co-operation in howler monkeys: an alternative model to explain organization in non-human primates [dissertation]. Urbana (IL), University of Illinois, 2007 http://hdl.handle.net/2142/85276

[45] Tesh R. B., Travassos da Rosa A. P. A., Guzman H., Araujo T. P., and Xiao S. Y.. Immunization with heterologous flaviviruses protective against fatal West Nile encephalitis. Emerg. Infect. Dis. 2002; 28:245–261. 10.3201/eid0803.010238 PMC2732478 11927020

[46] Halstead SB, Rojanasuphot S, Sangkawibha N. Original antigenic sin in dengue. Am J Trop Med Hyg. 1983; 32(1):154–6. 6824120 10.4269/ajtmh.1983.32.154

[47] Di Bitetti MS, Placci G, Brown AD, Rode DI. Conservation and population status of the brown howling monkey (*Alouattafuscaclamitans*) in Argentina. Neotropical Primates.1994; 2:1–4.

[48] Roberts DR, Peyton EL, Pinheiro FP, Balderrama F, Vargas R. Associations of arbovirus vectors with gallery forests and domestic environments in southeastern Bolivia. Bull PAHO. 1984; 18:337–350. 6525479

[49] de Thoisy B, Dussart P, Kazanji M. Wild terrestrial rainforest mammals as potential reservoirs for flaviviruses (yellow fever, dengue 2 and St Louis encephalitis viruses) in French Guiana. Trans Roy Soc Trop Med Hyg. 2004; 98:409–412. 10.1016/j.trstmh.2003.12.003 15138077

[50] Hanley KA, Monath TP, Weaver SC, Rossi SL, Richman RL, Vasilakis N. Fever versus fever: the role of host and vector susceptibility and interspecific competition in shaping the current and future distributions of the sylvatic cycles of dengue virus and yellow fever virus. Infect Genet Evol. 2013;19:292–311. 10.1016/j.meegid.2013.03.008 23523817 PMC3749261

[51] Pauvolid-Corrêa A, Kenney JL, Couto-Lima D, Campos ZM, Schatzmayr HG, Nogueira RM, et al Ilheus virus isolation in the Pantanal, west-central Brazil. PLoSNegl Trop Dis. 2013; 7: e2318. 10.1371/journal.pntd.0002318 PMC3715421 23875051

